# Genomic Analysis of West Nile Virus Lineage 1 Detected in Mosquitoes during the 2020–2021 Outbreaks in Andalusia, Spain

**DOI:** 10.3390/v15020266

**Published:** 2023-01-17

**Authors:** María José Ruiz-López, Milagros Muñoz-Chimeno, Jordi Figuerola, Ana M. Gavilán, Sarai Varona, Isabel Cuesta, Josué Martínez-de la Puente, Ángel Zaballos, Francisca Molero, Ramón C. Soriguer, Maria Paz Sánchez-Seco, Santiago Ruiz, Ana Vázquez

**Affiliations:** 1 Estación Biológica de Doñana—CSIC, Avda. Américo Vespucio 26, 41092 Sevilla, Spain; 2 CIBER de Epidemiología y Salud Pública (CIBERESP), 28029 Madrid, Spain; 3 Centro Nacional de Microbiología, Instituto de Salud Carlos III, Majadahonda, 28220 Madrid, Spain; 4 Unidad Bioinformática, Unidades Centrales Científico-Técnicas, Instituto de Salud Carlos III, 28220 Madrid, Spain; 5 Escuela Internacional de Doctorado de la UNED (EIDUNED), Universidad Nacional de Educación a Distancia (UNED), 28232 Madrid, Spain; 6 Departamento de Parasitología, Universidad de Granada, Campus de Cartuja s/n, 18071 Granada, Spain; 7 Unidad Genómica, Unidades Centrales Científico-Técnicas, Instituto de Salud Carlos III, 28220 Madrid, Spain; 8 CIBER de Enfermedades Infecciosas (CIBERINFEC), 28029 Madrid, Spain; 9 Servicio de Control de Mosquitos de la Diputación Provincial de Huelva, Ctra. Hospital Infanta Elena s/n, 21007 Huelva, Spain

**Keywords:** *Culex*, complete genome, flavivirus, vector-borne diseases, zoonosis

## Abstract

Emerging infectious diseases are one of the most important global health challenges because of their impact on human and animal health. The vector-borne West Nile virus (WNV) is transmitted between birds by mosquitos, but it can also infect humans and horses causing disease. The local circulation of WNV in Spain has been known for decades, and since 2010, there have been regular outbreaks in horses, although only six cases were reported in humans until 2019. In 2020, Spain experienced a major outbreak with 77 human cases, which was followed by 6 additional cases in 2021, most of them in the Andalusian region (southern Spain). This study aimed to characterize the genomes of the WNV circulating in wild-trapped mosquitoes during 2020 and 2021 in Andalusia. We sequenced the WNV consensus genome from two mosquito pools and carried out the phylogenetic analyses. We also compared the obtained genomes with those sequenced from human samples obtained during the outbreak and the genomes obtained previously in Spain from birds (2007 and 2017), mosquitoes (2008) and horses (2010) to better understand the eco-epidemiology of WNV in Spain. As expected, the WNV genomes recovered from mosquito pools in 2020 were closely related to those recovered from humans of the same outbreak. In addition, the strain of WNV circulating in 2021 was highly related to the WNV strain that caused the 2020 outbreak, suggesting that WNV is overwintering in the area. Consequently, future outbreaks of the same strain may occur in in the future.

## 1. Introduction

The flavivirus West Nile virus (WNV), is one of the most relevant emerging vector-borne pathogens and the most important causative agent of viral encephalitis worldwide [1]. It thus has a considerable impact both on public and animal health [2]. WNV is maintained in nature in an enzootic cycle involving ornithophilic mosquitoes, which are transmission vectors, and several species of birds that act as reservoirs [3]. Sometimes, infected mosquitoes bite other vertebrates, including mammals, producing a WNV spill-over that in some species like humans and horses might lead to neuroinvasive disease. WNV has been described in all continents except Antarctica and its presence in Europe has been known since 1962 [4]. Initially, its presence was considered the result of periodic introductions by migratory birds from Africa (reviewed in [5]). However, in the early 2000s, the incidence across Europe started to increase and it became clear that the virus may be endemic in several European countries, raising public health concerns [6].

WNV is a positive-sense, single-stranded RNA virus, and as such, it has an extraordinary geographic and temporal variability. So far, several WNV genetic lineages have been proposed based on phylogenetic analysis, and different strain genome sequences can differ from each other by more than 20–25% [7]. As a result, many WNV variants have evolved independently in different parts of the world and can adapt to local transmission cycles while they circulate and spread in different regions [3]. Among the different lineages in Europe, lineages 1 and 2 have been associated with outbreaks in humans. Lineage 1 is distributed throughout the world and consists of two clades: 1a (found in Africa, Europe, Middle East, Asia, and America), and 1b (Australian Kunjin virus) [8]. Lineage 2 was traditionally found in sub-Saharan Africa, but most recently, it has been spreading from Eastern towards southern Europe (including Spain in 2017 and 2020) [9].

Records of the presence of WNV in Spain derived, among others, from seroprevalence studies [10,11] and virus detection in birds [12] and mosquitoes [13,14]. In 2007, the virus was isolated for the first time from two Real eagles in Toledo, central Spain [15]. In horses, antibodies were first detected in 2005 in Doñana National Park in Andalusia [16], with the first outbreak in horses being reported in 2010. Since then, every year there have been outbreaks in horses in the country. Cases in humans occurred in the south-west region of the country: the first case was detected in Badajoz in 2004 [17], two cases were detected in Cadiz in 2010 [18], and three in Seville in 2016 [19]. In the summer of 2020, Spain experienced the biggest WNV human outbreak in this area with 77 infected and eight deaths [19]. This has been the largest outbreak of a mosquito borne disease in Spain since the eradication of malaria in 1964. Again, in 2021, there were six human cases in this area, including one fatal case (ECDC 2022. Epidemiological update: WNV transmission season in Europe, 2021 [20]).

Overall, three different lineages have been reported in Spain. In 2006, the putative lineage 6 was detected in mosquitoes from Andalusia [13] and, since 2008, lineage 1 has been repeatedly identified in mosquitoes, horses and birds, mainly in the south-west of Spain. More recently, lineage 2 was reported in 2017 and 2020 in Catalonia (north-east Spain; [9]). Genetic analyses of sequences of lineage 1 suggest that several independent introductions have occurred during the XXI century [21]. The increased number of human cases recorded in 2020 and 2021 led to speculation on the possible introduction of a more pathogenic strain. Our aim here was to report the full genomes and characterize the WNV lineage 1 circulating in mosquitoes in 2020 and 2021 in southern Spain, after the two main outbreaks in humans reported in these consecutive years. We also compared the genome sequences found in mosquitoes with the genomes sequenced from human samples obtained during the outbreak and the complete sequences available from previous years from mosquitoes, horses, and birds to better understand the WNV eco-epidemiology in Spain. 

## 2. Materials and Methods

### 2.1. Mosquito Surveillance and Identification

Mosquitoes were captured in different localities of the provinces of Huelva and Seville (Andalucia, Spain) during 2020 and 2021. In each locality, we placed three BG traps that operated for 24 h and were baited with approximately 1 kg of dry ice each to generate a continuous flow of CO_2_ at the entrance of the trap. Mosquitoes were transported in dry ice to the lab and stored at −80 °C before further processing. Mosquitoes were identified following identification keys [22,23] and pooled in groups of up to 50 individuals by species, sex, sampling site, date, and locality. In total we tested 419 pools of *Cx. perexiguus* females in 2020 (see [24] for further details), and 1024 in 2021. 

### 2.2. Molecular Diagnosis of WNV

Before RNA extraction, pools were homogenized in sterile MEM buffer (minimum essential buffer) supplemented with 10% bovine fetal serum, 0.5% of penicillin, and streptomycin and 10% L-Glutamine (Sigma-Aldrich, St. Louis, MO, USA). Viral RNA was extracted from mosquito pools or cell culture supernatants by using a QIAamp Viral RNA Mini Kit (QIAGEN, Valencia, CA, USA). The presence of WNV was tested using a one-step real-time reverse-transcription qRT-PCR targeting a conserved sequence of the 3′-UTR region of the WNV genome and using an internal control to avoid false negatives [25]. qRT-PCRs were carried out on the LightCycler 480 (ROCHE, Basel, Switzerland) and 7500 Fast (Applied Biosystem, Waltham, MA, USA). 

### 2.3. Viral Sequencing and Genome Assembly

**Figure 1:**
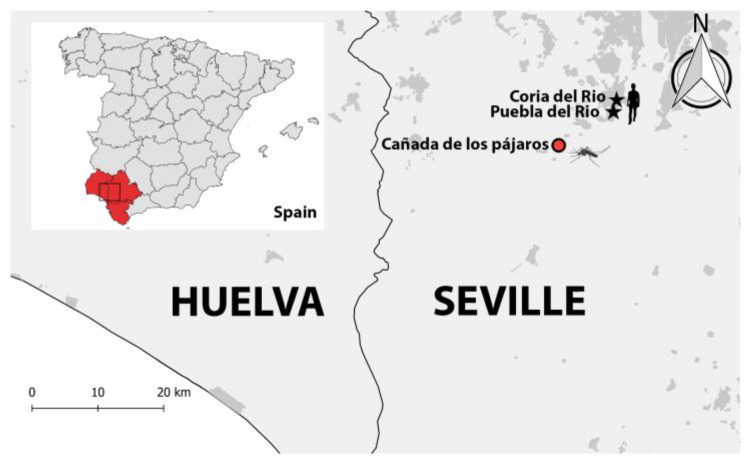
Map showing the three provinces of Andalusia where human and equine cases occurred during 2020 and 2021 (Huelva, Seville and Cadiz). In the main map, the stars represent the two sites in Seville that concentrated most of the cases during 2020 and 2021 season. The red circle is the location where the mosquito pools used for whole genome sequencing were captured. The dark gray areas are the cities and villages.

The qRT-PCR identified 33 *Cx. perexiguus* positive pools (7.88%) in 2020, and 106 (10.35%) positive pools in 2021. For both years, we selected two positive pools of *Cx. perexiguus* (one per year) with a Ct lower than 25 in the qRT-PCR. We cultured these pools in Vero cells to carry out WNV isolation. These samples were also selected because they were collected at the sampling site “Cañada de los pájaros” (Seville, Andalusia, Spain), which belongs to the municipality of Puebla del Rio where most of the human cases occurred (Figure 1). Complete genome sequences were obtained for the WNV isolates after one passage in Vero cells. The library was prepared using a metagenomics approach. Briefly, viral RNA was extracted using the Quick-RNA Viral kit (Zymo, Irvine, CA, USA). RNA was quantified and verified using a Bioanalyzer 2100 with RNA 6000 Nano Kit (Agilent Technologies, Santa Clara, CA, USA). Sample library preparation was conducted using the NEBNext^®^ Ultra II RNA Library Prep Kit for Illumina^®^ with NEBNext^®^ Multiplex Oligos for Illumina^®^, Index Primers Set 3 (New England BioLabs Inc., Ipswich, MA, USA) and sequenced on an Illumina MiSeq v2 (300 Cycles). 

Sequencing samples were analyzed for viral consensus genome reconstruction using viralrecon pipeline v2.4.1 (https://github.com/nf-core/viralrecon) (accessed on 24 January 2022) [26] written in Nextflow (https://www.nextflow.io/) (accessed on 24 January 2022) in collaboration between the nf-core (https://nf-co.re/) (accessed on 24 January 2022) community and the Bioinformatics Unit of the Institute of Health Carlos III (BU-ISCIII) (https://github.com/BU-ISCIII) (accessed on 24 January 2022). In this pipeline, fastq files containing raw reads were first analyzed for quality using FastQC v0.11.9 [27]. Raw reads were trimmed using fastp v.0.23.2 [28], where a sliding window quality filtering approach was performed, scanning the reads with a 4-base wide sliding window, cutting 3′ and 5′ end bases when the average quality per base drops below a Qphred33 of 30. Reads shorter than 50 nucleotides and reads with more than 10% of read quality under Qphred 30 were removed. Additionally, poly-X sequences were removed from read ends. Trimmed reads were mapped against the reference WNV genome (JF719067.1) with bowtie2 v.2.4.4 [29], Picard v.2.26.10 (https://github.com/broadinstitute/picard) (accessed on 24 January 2022) and SAMtools v.1.14 [30] were used to generate viral genome mapping stats. To obtain statistics about the host genome content, we used Kraken2 v.2.1.2 [31] to run kmer-based mapping of the trimmed reads against the African green monkey (*Chlorocebus sabaeus*; TaxID 60711, GCF_015252025.1) and mosquito genome references *Culex quinquefasciatus* (TaxID 7176, GCF_015732765.1) and *Culex pipiens* (TaxID 7175, GCF_016801865.1).

Variant calling was carried out using ivar variants v.1.3.1 [32], which calls for low and high frequency variants from which variants with an allele frequency higher than 75 were kept to be included in the consensus genome sequences. Both variants included or not in consensus genome sequence were annotated using SnpEff v.5.0.e [33] and SnpSift v.4.3 [34]. Finally, BEDtools v2.30.0 [35] was used to obtain the viral genome consensus with filtered variants and mask genomic regions with coverage values lower than 10×. Final summary reports were created using MultiQC v.1.9 [36].

### 2.4. Phylogenetic Analysis

For the phylogenetic analyses, we selected the polyprotein region of the two sequenced genomes and compared them with 55 complete genomes from WNV lineage 1 available at Genebank. Multiple alignments were carried out with Clustal W, and the phylogenetic tree was generated using a maximum likelihood approach and a general time reversible model (GT+G+I) and 1000 bootstraps in MEGA11: Molecular Evolutionary Genetics Analysis version 11 [37]. We also estimated the genome distance and compared the aminoacid changes between the sequences obtained here with the four human sequences from 2020 and previous complete sequences from this region of previous years. 

## 3. Results

Here, we used a metagenomic approach to obtain the genome of WNV detected from two pools of mosquitoes (hereafter 20c124 and 21C560) captured in 2020 and 2021, respectively. For both samples, we obtained more than 2 million reads per sample, of which 22% and 13% belonged to WNV. The WNV genomes of the two samples were covered 100% with a depth of coverage > 10×. The median depth of coverage of the genome was 5985 and 3411, respectively. Both viral consensus genome sequences have more than a hundred variants with respect to the reference genome (139 and 144), being 12 of them in both cases missense variants. Further details can be found in Table 1. 

**Figure 2:**
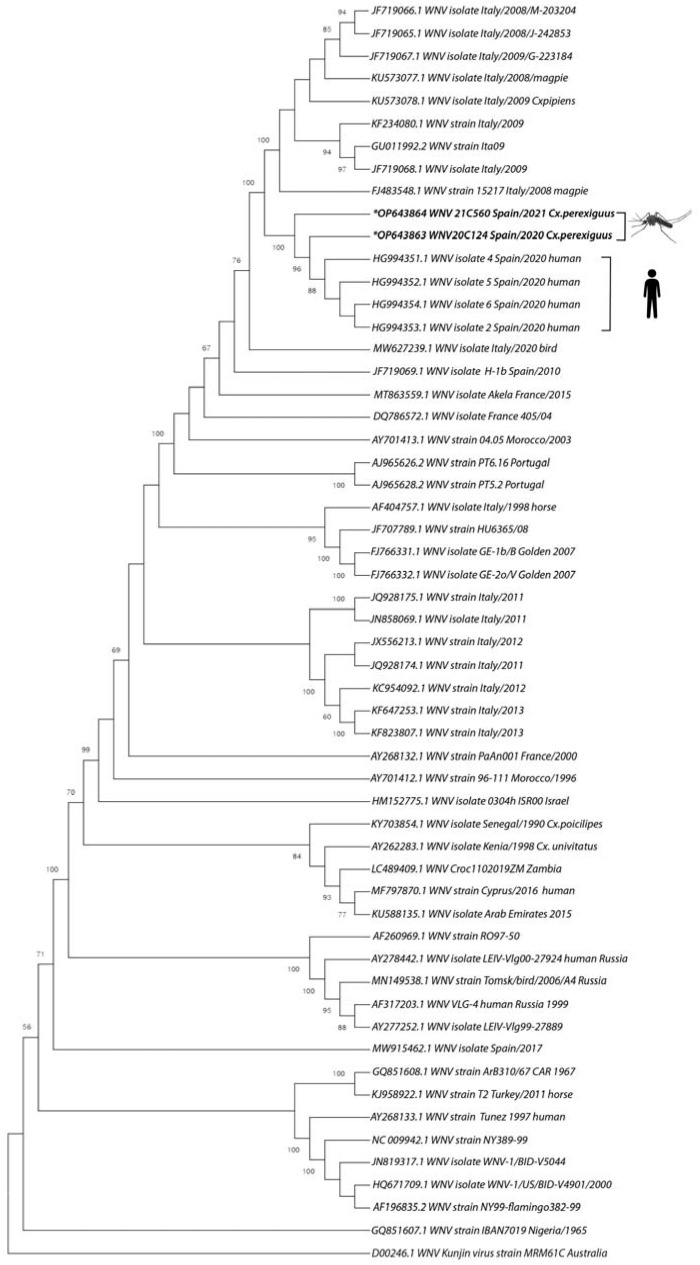
Phylogenetic analysis of polyprotein nucleotide sequences of WNV-L1. The tree was constructed using a maximum likelihood method. Bootstrap values are given for 1000 replicates. Viral sequences are identified by the GenBank accession number, country and year of isolation.

Maximum likelihood phylogenetic analyses on the polyprotein gene confirmed that both sequences obtained here belonged to the WNV lineage 1, clade 1a, cluster 2, Mediterranean subtype [21,38] (Figure 2). Both genomes clustered together with the four Spanish genomes of WNV isolated from human samples in 2020 (Figure 2). The genome sequenced from the sample collected in 2020 (20c124, GenBank Accession number OP643863) is more closely related with the human samples (mean distance estimation of 0.0006 or 0.06% divergence) than the sample collected in 2021 (21C560, GenBank Accession number OP643864; mean distance estimation of 0.0022 or 0.22% divergence). The mean distance between 20c124 and 21C560 was 0.0019 (0.19% divergence). The closest genomes to this group are from WNV samples obtained in 2008 and 2009 in Italy, while the sample collected in Spain in 2010 from a horse, in 2008 from a pool of mosquitoes and 2007 from a bird diverge at a previous branching point. The sequences obtained from a bird in 2017 does not group with the new sequences either, confirming that both belong to different clusters. The amino acid analysis shows that the Spanish mosquito isolates 20c124 and 21c560 and the four human sequences shared 5 unique amino acids changes in the polyprotein when we compared them with the most related sequence obtained from Italy (JF719067.1) and the rest of Spanish sequences obtained in previous years (Supplementary Materials). Remarkably, each sequence obtained in this work showed only one unique amino acid substitution, A50V (20c124) in the envelope and A177V (21c560) in the NS3. We were not able to conduct further analyses because available human sequences are incomplete in some parts of the genome. 

## 4. Discussion

The metagenomic approach used in this study allowed us to directly generate genomic sequences of WNV, which is crucial for phylogenetic analysis if this is used for detailed outbreak investigation. Because we are working with pools of mosquitoes, the genomes were generated by creating consensus genomes. 

The results of this study strongly support that WNV circulating on 2021 was highly related with the WNV strain that caused the outbreak during the year 2020. This supports the possibility that WNV is overwintering in the area and, consequently, future outbreaks of the same strain are likely to occur. Historically, it was claimed that the source of WNV introduction in Europe were African countries through the migration of infected birds [39]. However, the analyses of viral genomes has allowed better study of the evolution of WNV circulating strains, revealing that most outbreaks that have occurred in Europe are the result of a limited number of introductions and that dispersal within the continent is more important than previously assumed [5,40]. In fact, several studies have found examples of overwintering WNV lineage 2 [9,41]. Regarding the origin of the WNV strain found in Spain in 2020 and 2021, two different scenarios were proposed by Casimiro-Soriguer et al., (2021) [42]: (i) an endemic strain in the Mediterranean area that produces outbreaks in Spain and Italy, and (ii) an endemic Italian reservoir from which the virus has been introduced several times in Spain. Although further studies are needed with more viral genome sequences from mosquitos sampled at different time points, our results support the first scenario, with an Italian-related strain showing endemic circulation in southern Spain. This also shows that the recent increase of WNV human cases in 2020 and 2021 in the studied area was not explained by different virus introductions.

Our results also showed that both genomes clustered together with the four Spanish genomes of WNV isolated from human samples in 2020. Although the collection site of the mosquito pools was carried out in a natural area, this sample site is only 8 to 12 km a from the epicenter of the human outbreak (the municipalities of Coria and Puebla de Rio). Three of the human genomes sequenced come from this area, confirming the intimate connection between the circulation of WNV in *Cx. perexiguus* in natural areas and the outbreaks in urban areas. 

In conclusion, access to complete genomes of the WNV strains circulating in endemic areas is important to better understand WNV spread in Europe and its eco-epidemiology. Mosquito surveillance can play a key role in enabling WNV genome sequencing and allow, in short periods of time, the characterization of the genomes of the lineages circulating. This will increase our capacity to understand virus amplification and dispersal across the continent. This type of study is particularly relevant right now in Europe in general, and particularly in Spain, due to the cocirculation of different strains and the recent expansion of WNV lineage 2 [21]. In addition, a recent study showed that different strains are potentially being introduced [9], complicating the scenario and highlighting the importance of genomic monitoring as a part of the surveillance plan. 

## Figures and Tables

**Table 1:** Raw sequencing data, where the host are Vero Cells, and the reference sequence is JF7190.67.

Sample	20c124	21c560
Total reads	2,225,818	2,126,416
Reads host R1	865,285	920,637
Reads host	1,730,570	1,841,274
%reads host	77.75	86.59
Reads virus	475,496	275,042
%reads virus	21.36	12.93
Unmapped reads	19,752	10,100
%unmaped reads	0.89	0.47
medianDPcoverage virus	5985	3411
Coverage > 10×(%)	100	100
Variants in consensus ×10	139	144
Missense Variants	12	12
%Ns10×	0.00	0.00

## Data Availability

Genome sequences are available in GenBank.

## Supplementary Materials

The following supporting information can be downloaded at: https://www.mdpi.com/article/10.3390/v15020266/s1, Table S1: Comparison of the amino acid substitutions between the isolates detected in this work and the strains detected in Spain in previous years and the related Italian sequence JF719067.1 (used as reference) which belong to the WNV lineage 1 cluster 2.
