# Supplementary material for: Genomic Analysis of West Nile Virus Lineage 1 Detected in Mosquitoes during the 2020–2021 Outbreaks in Andalusia, Spain
Source: Viruses. 2023 Jan 17;15(2):266. doi: 10.3390/v15020266 (PMC9962355; doi:10.3390/v15020266)
Supplement: Supplementary file 1 [file viruses-15-00266-s001.zip › viruses-2150577-supplementary.pdf]

Supplementary Material

Table S1. Comparison of the amino acid substitutions between the isolates detected in this work and the strains detected in Spain in previous years and the related Italian sequence JF719067.1 (used as reference) which belong to the WNV lineage 1 cluster 2. The amino acids positions were calculated from the complete sequence AF404757. * stop codon. ? no sequence available. The unique amino acid from the sequences obtained in this work are in red. The unique amino acids shared between the human sequences and the obtained from mosquitoes in this work are in bold.

Viral Poliprotein	Amino Acid Position	JF719067 Italy/2009 Gull	FJ766331 GE1b/B Spain/2007 Golden	FJ766332 GE2o/V Spain/2007 Golden	JF707789 HU6365/08 Spain/2008 mosquito	JF719069 H-1b Spain/2010 Horse	MW915462 NG-b Spain/2017 Goshawk	HG994351 isolate 4 Spain/2020 Human	HG994352 isolate 5 Spain/2020 Human	HG994354 isolate 6 Spain/2020 Human	HG994353 isolate 2 Spain/2020 Human	20c124 Spain/2020 mosquito	21c560 Spain/2020 mosquito
C	34	M	V	V	V	-	-	-	-	-	-	-	-
	**52**	A	-	-	-	-	-	**T**	**T**	**T**	**T**	**T**	**T**
	109	T	-	-	-	A	-	?	?	?	?	-	-
	110	G	-	-	R	-	-	?	?	?	?	-	-
M	2	L	-	-	V	-	-	-	-	-	-	-	-
	64	V	-	-	-	-	A	-	-	-	-	-	-
E	24	V	-	-	-	I	-	-	-	-	-	-	-
	50	A	-	-	-	-	-	?	-	?	-	**V**	-
	51	A	T	T	T	-	-	-	-	-	-	-	-
	88	P	S	S	S	-	-	-	-	-	-	-	-
	352	V	-	-	-	-	-	M	-	-	-	-	-

	479	R	-	-	-	-	-	-	-	-	K	-	-
	481	I	-	-	V	-	-	-	-	-	-	-	-
	35	Y	H	H	H	-	-	-	-	-	-	-	-
	70	S	-	-	-	-	A	-	-	-	-	-	-
	**170**	K	-	-	-	-	-	**R**	**R**	**R**	**R**	**R**	**R**
**NS1**	208	D	H	H	H	-	-	-	-	-	-	-	-
	**284**	M	T	T	T	T	T	T	T	T	T	T	T
	289	E	G	G	G	-	-	-	-	-	-	-	-
	338	Q	-	-	-	R	-	-	-	-	-	-	-
	119	H	-	-	-	-	Y	-	-	-	-	-	-
	142	I	-	-	V	-	-	-	-	-	-	-	-
**NS2A**	158	L	-	-	-	V	-	-	-	-	-	-	-
	187	I		-	-	-	V	-	-	-	-	-	-
	**211**	G	-	-	-	-	-	**R**	**R**	**R**	**R**	**R**	**R**
**NS2B**	97	I	-	-	-	-	L	-	-	-	-	-	-
	107	I	-	-	-	-	V	-	-	-	-	-	-
	143	N	-	-	-	-	S	-	-	-	-	-	-
	177	A	-	-	-	-	-	-	-	-	-	-	**V**
	249	T	P	P	-	-	-	-	-	-	-	-	-
	253	N	-	-	-	-	S	-	-	-	-	-	-
**NS3**	338	T	-	-	S	-	-	-	-	-	-	-	-
	436	T	-	-	-	-	A	A	A	A	A	A	A
	453	A	-	-	I	-	-	-	-	-	-	-	-
	465	N	-	-	-	S	-	-	-	-	-	-	-
**NS4A**	6	I	-	-	-	-	V	-	-	-	-	-	-
	85	I	V	V	V	V	V	V	V	V	V	V	V
	100	S	P	P	P	P	P	P	P	P	P	P	P
**NS4B**	17	F	-	-	-	-	-	L	-	-	-	-	-
	23	V	-	-	-	A	-	-	-	-	-	-	-
	115	A	T	T	T	-	-	-	-	-	-	-	-

	241	T	-	-	-	-	I	-	-	-	-	-	-
	249	D	-	-	-	-	E	-	-	-	-	-	-
**NS5**	**41**	K	-	-	-	-	-	**R**	**R**	**R**	**R**	**R**	**R**
	88	C	-	-	-	S	-	-	-	-	-	-	-
	194	K	-	-	R	-	-	-	-	-	-	-	-
	258	A	V	V	V	V	V	V	V	V	V	V	V
	422	K	R	R	R	R	R	-	-	-	-	-	-
	426	E	A	A	-	-	-	-	-	-	-	-	-
	436	M	I	I	I	-	-	-	-	-	-	-	-
	450	H	-	-	-	-	Y	-	-	-	-	-	-
	503	K	-	-	-	-	Q	-	-	-	-	-	-
